# Tumor suppressive effect of scavenger receptor class A member 5 overexpression in colorectal cancer by regulating PI3K/AKT/mTOR pathway

**DOI:** 10.1007/s13258-021-01139-3

**Published:** 2021-08-21

**Authors:** Yi Li, Feng Peng, Xiangyun Tan, Jin Wang, Yeqing Xu

**Affiliations:** Department of General Surgery, The Affiliated Zhuzhou Hospital Xiangya Medical College CSU, No. 116, South Changjiang Road, Tianyuan District, Zhuzhou, 412007 Hunan China

**Keywords:** Scavenger receptor class A member 5, Colorectal cancer, PI3K/AKT/mTOR pathway, CRC xenograft mice

## Abstract

**Background:**

Colorectal cancer (CRC) exhibits high risks of morbidity and mortality.

**Objective:**

To investigate the effect of scavenger receptor class A member 5 (SCRAR5) on CRC and its mechanism on modulation of cancer development.

**Methods:**

The SCRAR5 expression in four kinds of CRC cell lines (SW620, SW480, HT29, and HCT116) was measured by quantitative PCR and western blotting, respectively. The effects of SCRAR5 abnormal expression on cell proliferation, apoptosis, and migration were analyzed by CCK-8 assay, EdU assay, colony-forming assay, flow cytometry assay, Transwell assay and wound healing assay, respectively. Meanwhile, the involvements of PI3K/AKT/mTOR pathway with the role of SCRAR5 were investigated by western blotting. Afterwards, the in vivo effects of SCRAR5 abnormal expression on CRC xenograft mice were finally investigated by evaluating tumor volume, apoptosis and Ki67 expression.

**Results:**

SCRAR5 was lowly expressed in CRC cell lines, especially SW480 cells. Up-regulation of SCRAR5 significantly promoted cell apoptosis, reduced cell proliferation and migration in SW480 cells. Notably, SCRAR5 overexpression obviously inhibited the phosphorylation levels of PI3K, AKT, and mTOR. Reversely, SCRAR5 silence exhibited promoting effects on HT29 cells. Consistently, in vivo experiments also revealed that SCRAR5 overexpression remarkably suppressed tumor volume and Ki67 expression, as well as promoted cell apoptosis.

**Conclusions:**

Overall, up-regulating of SCRAR5 obviously inhibited CRC tumor growth in vitro and in vivo, which might be related to PI3K/AKT/mTOR pathway.

## Introduction

As the common digestive system malignant tumors, and colorectal cancer (CRC) exhibits high risks of morbidity and mortality (Keum and Giovannucci 2019; Siegel et al. 2020). Currently, the conventional treatment methods for CRC contain chemotherapy, surgical resection, and personalized pharmacological treatments (Mármol et al. 2017; Rejhová et al. 2018). Although the diagnosis and treatments for CRC have the intensive progress, failed chemotherapy due to drug resistance to chemotherapeutic drugs, higher metastasis and complex pathogenesis result in unsatisfactory prognosis (Mármol et al. 2017). Therefore, the underlying pathogenesis should be investigated to uncover novel and effective treatment targets for CRC.

Over the last few decades, increasing researchers investigate the prognosis biomarkers in order to search for the effective treatment methods for cancers (Shukla 2017). The research and development of antitumor drug targeting prognosis biomarkers has been focused for treating cancer (Wang et al. 2019). Scavenger receptors (SRs) as a cluster of heterogeneous molecules present at the phagocytes surface bind to various ligands such as lipids, proteins, polysaccharides, polyribonucleotides, and modified lipoproteins, thereby enhancing the elimination of harmful non-self or altered-self targets (Gusev et al. 2020). Previous study has demonstrated that adhesion, endocytosis, phagocytosis and signal transduction are involved in the clearance of harmful substances (Canton et al. 2013). SRs can be divided into different classes, including scavenger receptor class A member 1 (SCRAR1), SCRAR2 (also named as macrophage receptor with collagenous domain), SCRAR3, SCRAR4, SCRAR5, according to the diverse structure and biological function (PrabhuDas et al. 2017). Notably, SCRAR5 is a newly identified SR that similar to the SCRAR1 and SCRAR2 in the amino acid sequence; however, they exhibit different ligand binding activities (Whelan et al. 2012). SCARA5 is able to deliver non-transferrin bound iron to the kidney which ensures the development of the kidneys, as well as involves with adipogenesis and connective tissue homeostasis (Whelan et al. 2012; Yu et al. 2020a). SCRAR5 is widely expressed in ovary, lung, skin, adrenal gland, kidney, bladder, and testis (Plüddemann et al. 2007). SCARA5 is coded by gene locating on chromosome 8p21 which usually disappears in cancers (Yan et al. 2012). Accumulated studies have demonstrated the abnormal expression of SCARA5 in several cancers, including lung Adenocarcinoma (Yu et al. 2020b), breast cancer (Mamoor 2020), hepatocellular carcinoma (Liu et al. 2018a), and oral squamous cell carcinoma (Liu et al. 2018c). Interestingly, recent study based on bioinformatics analysis has shown that compared with normal tissues, SCARA5 is lowly expressed in CRC clinical specimens (Liu et al. 2020). However, little research has investigated the role mechanism of SCARA5 in CRC.

In the current research, the SCARA5 expression were firstly detected in various CRC cell lines. Then, the effects of SCARA5 abnormal expression on cell proliferation, apoptosis and migration in CRC cells and CRC xenograft mice were unraveled. It is well-known that the mammalian target of rapamycin (mTOR) and phosphatidylinositol-3-kinase (PI3K)/Akt pathways are considered as crucial pathways to regulate cell survival both in pathological and physiological conditions (Ersahin et al. 2015). Accumulating evidence has demonstrated that targeting PI3K/Akt/mTOR pathway is a potential therapeutic strategy for cancers (Polivka Jr and Janku 2014; Porta et al. 2014). Thus, we explored whether PI3K/Akt/mTOR pathway participates in the effects of SCARA5 on CRC.

## Materials and methods

### Cell culture

Human normal colonic epithelial cell line HCoEpic and four CRC cell lines, including SW620, SW480, HT29, and HCT116, were purchased from Nanjing KeyGen Biotech. All the cell lines were stored in liquid nitrogen and recovered in complete DMEM medium (Thermo, China) before use. Then, these cells were cultured in humidified chamber with 5% carbon dioxide at 37 °C.

### Animal experiments

This study was approved by Experimental Animal Welfare Ethics Committee of Central South University before experiments. Healthy nude mice (weighted 18–22 g) were obtained and randomly assigned to five groups. Mice (n = 5, each group) were treated with PBS (control group), OE-SCRAR5, OE-NC, shSCRAR5, and shNC via rapid tail vein injection. Then, to obtain the mouse xenograft model, 1 × 10^6^ of SW480 cells per mouse were subcutaneously inoculated in mice. The tumor volume was measured every 1 weeks for 4 weeks. At fourth weeks, the tumor from mice was obtained for the following experiments.

### Cell transfection

For analysis of the impacts of SCRAR5 abnormal expression on CRC cells, SCRAR5 overexpression plasmid (OE-SCRAR5), SCRAR5 silence plasmid (shSCRAR5), and the corresponding negative controls OE-NC, and shNC were obtained from GenePharma (Shanghai, China). The SW480 or HT29 cells were transiently transfected with these plasmids, respectively, using the Lipofectamine™ 3000 transfection reagent (Thermo).

### Cell viability

The viabilities of SW480 and HT29 cells that underwent the above treatments were evaluated by cell counting kit-8 (CCK-8, Beyotime). Briefly, SW480 or HT29 cells were inoculated to the 96-well plates, and underwent the above treatments for another 24, 48 or 72 h, which were then reacted with CCK-8 at room temperature for 2 h. The absorbances at 450 nm (OD450) were finally detected by the micro-plate reader. At least three biological replicates of the CCK-8 assay were performed for evaluating SW480 and HT29 cell viability.

### 5-Ethynyl-2′-deoxyuridine (EdU) assay

EdU kit (Invitrogen) was utilized to detect cell proliferation. Specifically, the SW480 and HT29 cells at the logarithmic growth phase underwent the above treatments for 48 h, and then cultured with EdU for 2 h. Next, 4% paraformaldehyde was used to fix cells, and cells were stained with Apollo and 4′,6-diamidino-2-phenylindole (DAPI), in turn, and mounted in glass slide. Lastly, the cells were observed using inverted microscope (Olympus, Japan).

### Colony-forming assay

Addition to EdU assay and CCK-8 assay, colony-forming assay was also utilized to evaluate cell proliferation. In brief, 400 cells per well of SW480 and HT29 cells were seeded in 6-well plates, followed by transfected with OE-SCRAR5, OE-NC, shSCRAR5, and shNC, respectively. After 14 days, cells underwent fixation and incubated with crystal violet. Lastly, the colonies were observed using light microscope (Olympus, Japan).

### Cell apoptosis assay

Annexin V-fluorescein isothiocyanate (FITC)/propidium iodide (PI) kit was used to observe cell apoptosis. The SW480 and HT29 cells were exposed the different treatments for 24 h and harvested by trypsin. After rinsed with PBS, cells were resuspended with buffer, followed by the exposure of Annexin V-FITC and PI. The treated cells were observed using flow cytometer (BD).

### Cell migration assay

Cell in vitro migration was evaluated using wound healing and transwell assay. For wound healing assay, SW480 and HT29 cells were seeded in 6-well plates and cultured until 60% of cell confluence. Subsequently, cells were scratched by pipette tips as a vertical lineation, and cultured in serum-free DMEM. Next, cells were transfected with OE-SCRAR5, OE-NC, shSCRAR5, and shNC, respectively. The wound area was measured at 0, 24 and 48 h by light microscope. For the transwell assay, the bottom compartment was added with complete culture medium. The transfected cells were inoculated in the Matrigel Matrix coated compartment with culture medium (serum deprivation) for 24 h. Followed by the cells fixation and staining with crystal violet, the migrated cells were observed by inverted microscope (Olympus, Japan).

### Quantitative RT-PCR

Total RNA samples were obtained from the transfected SW480 and HT29 cells by the Invitrogen TRIzol Reagent (Thermo) following the manufacturer’s instructions. Then, complementary DNA (cDNA) samples were prepared from the isolated RNA using the QuantiTect Reverse Transcription Kit (QIAGEN). The following quantitative PCR method was finished using the SYBR Premix Ex Taq TM II (Takara). GAPDH was used as the internal standards for mRNA quantitation. Sequences of primers were all presented in Table 1.

**Table 1:** Specific primers for qPCR assay

Gene	Primer sequence
SCARA5	Sense primer: 5′-TGTGGGCATCTTCATCTTAGC-3′
	Antisense primer: 5′-CTCTCATTCAGCCGGTTCAC-3′
GAPDH	Sense primer: 5′-GTGGATCAGCAAGCAGGAGT-3′
	Antisense primer: 5′-AAAGCCATGCCAATCTCATC-3′

### Western blotting

The transfected cells were lysed using lysis buffer (Beyotime, China) on ice to extract proteins. Then, proteins samples underwent resolving on PAGE gel and transferring to PVDF, followed by blocking the membrane and reacting with SCRAR5, phosphatidylinositol-3-hydroxykinase (PI3K), phosphorylated-PI3K (p-PI3K), AKT, p-AKT, mTOR, p-mTOR, or GAPDH primary antibody (1:1000, Abcam) at room temperature for 3 h. Second antibody (1:3000, Abcam) was then reacted with the membrane, and the expression of these proteins were observed using enhanced chemiluminescence (ECL, Millipore, USA).

### Tunel assay

Tumor tissues from different groups underwent formalin-fixation, paraffin embedding, and slice preparation. Then cell apoptosis in tumor tissues was evaluated by TUNEL staining using a commercial Kit (Roche, Mannheim, Germany) following the producer’s guideline. Image was photographed under a fluorescence microscope (Olympus, Tokyo, Japan).

### Immunohistochemistry (IHC)

The sections were obtained as described in Tunel assay. After deparaffinization and dehydration, the sections were treated with citrate buffer (pH 6.0), followed by the heat pretreatment at 80 °C and blocking with endogenous peroxide. Next, the sections were incubated with SCARA5 and Ki67 antibodies, respectively, followed by the incubation of second antibody. Ultimately, the sections were mounted with neutral resin and observed under a light microscope (Nikon, ECLIPSE CI).

### Statistical analysis

Quantitative data produced from at least biological repeats were analyzed for statistical significance with the SPSS 20.0 software. The Student *t* test and ANOVA were performed to evaluate differences between two and > 2 groups, respectively. A p value of < 0.05 was set as the threshold for significant differences.

## Results

### SCRAR5 expression was down-regulated in CRC cells

**Fig. 1:**
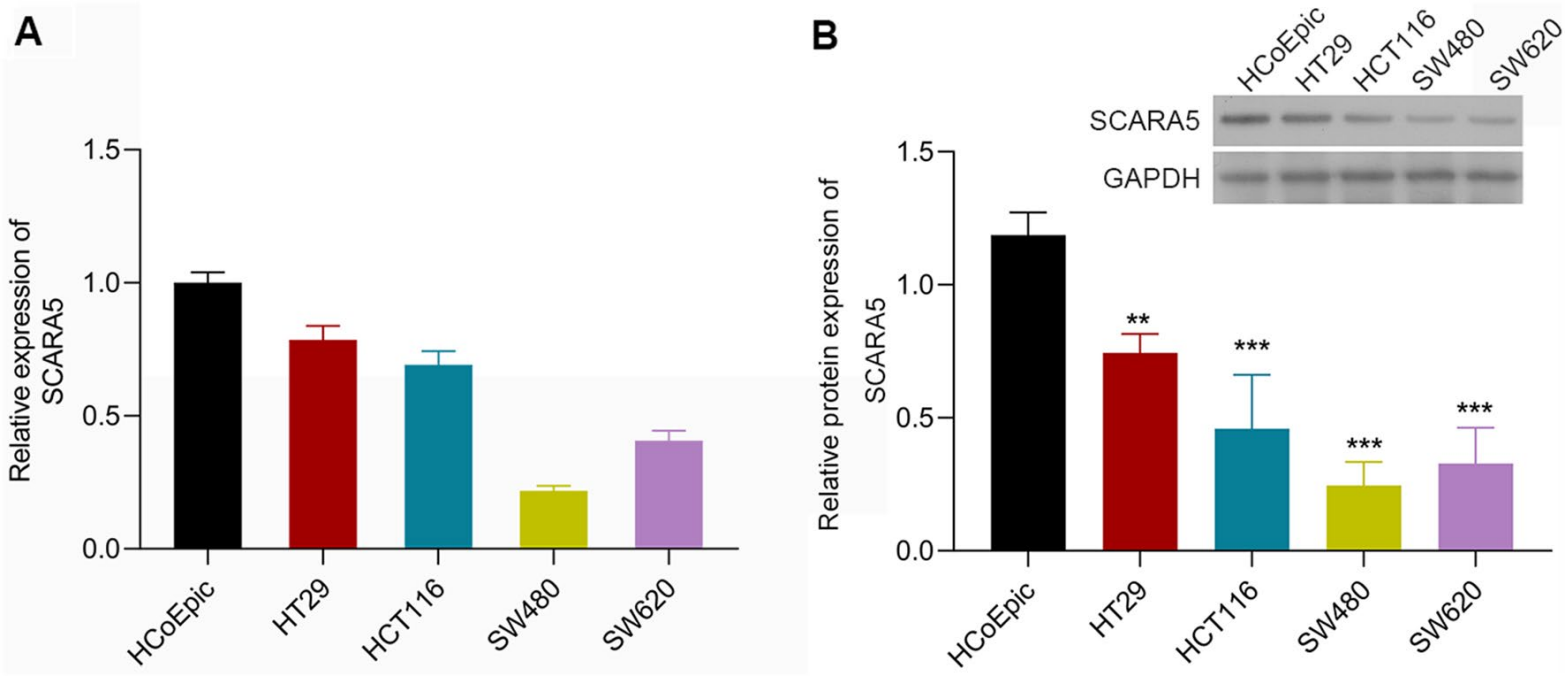
Scavenger receptor class A member 5 (SCRAR5) expression in colorectal cancer (CRC) cells. The mRNA and protein levels of SCRAR5 in CRC cell lines (HT29, HCT116, SW480, and SW620) was significantly lower than that in normal HCoEpic cell line by qPCR (**A**) and western blotting (**B**), respectively

Both the results of qPCR and western blotting showed obviously lower mRNA and protein levels of SCRAR5 in CRC cell lines (SW620, SW480, HT29, and HCT116) than that in normal HCoEpic cell line (Fig. 1A, B). Notably, SW480 and HT29 cell line, which had the lowest and highest expression of SCRAR5, respectively, were used for the further experiments.

### Effect of SCRAR5 abnormal expression on CRC cells

**Fig. 2:**
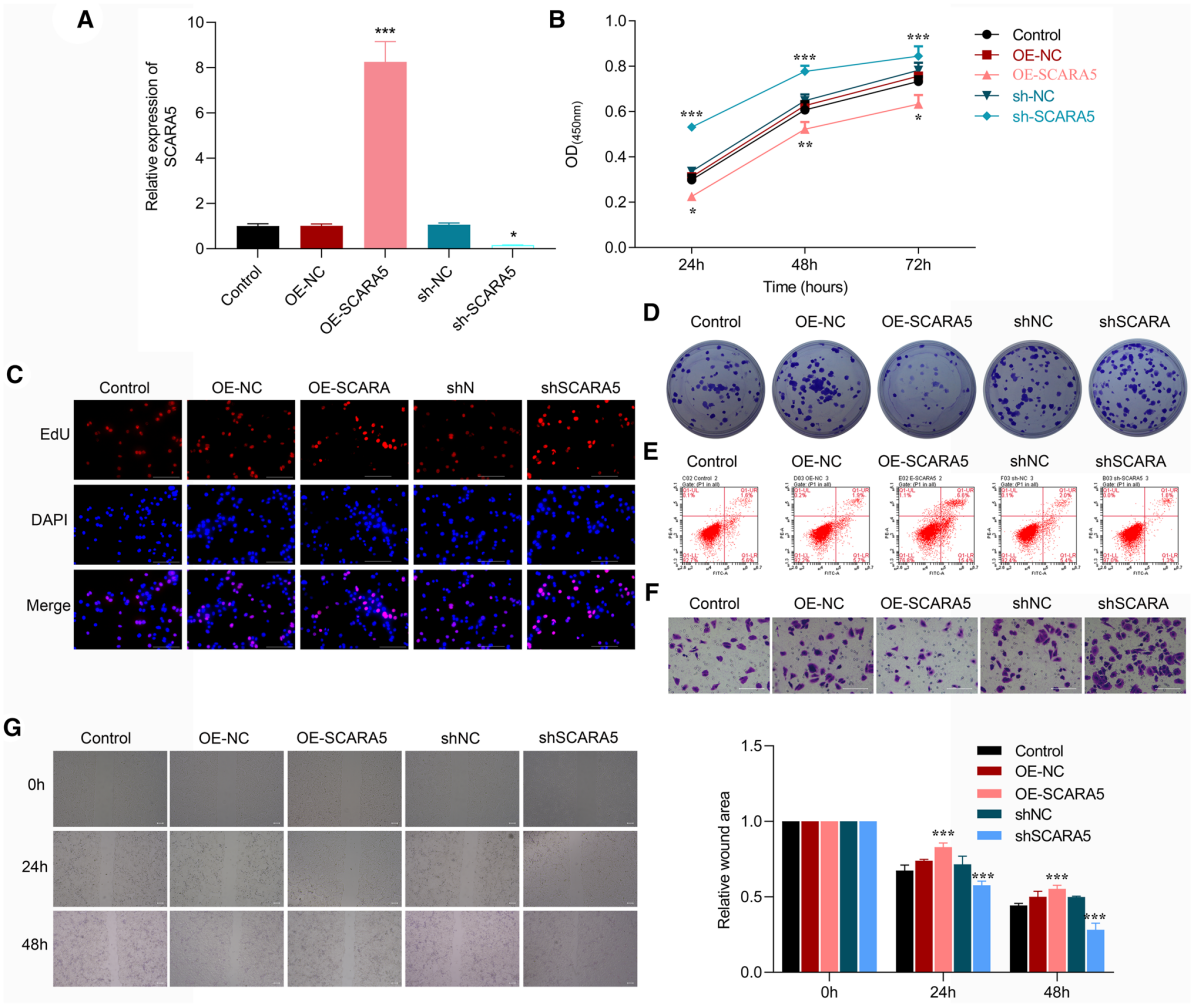
Scavenger receptor class A member 5 (SCRAR5) overexpression inhibited cell proliferation, induced cell apoptosis, and suppressed cell migration in SW480 cells. **A** The mRNA level of SCRAR5 in SW480 cells transfected with SCRAR5 overexpression plasmid (OE-SCRAR5), SCRAR5 silence plasmid (shSCRAR5), and the corresponding negative controls OE-NC, and shNC, respectively. **B** Cell viability of SW480 cells in control, OE-NC, OE-SCRAR5, shSCRAR5, and shNC groups, respectively, in time-dependent manner by CCK-8 assay. **C** Cell proliferation of SW480 cells in control, OE-NC, OE-SCRAR5, shSCRAR5, and shNC groups, respectively, by5-ethynyl-2′- deoxyuridine (EdU) assay. **D** The clone number of SW480 cells in control, OE-NC, OE-SCRAR5, shSCRAR5, and shNC groups, respectively, by colony-forming assay. **E** Cell apoptosis of SW480 cells in control, OE-NC, OE-SCRAR5, shSCRAR5, and shNC groups, respectively, by annexin V-fluorescein isothiocyanate (FITC)/propidium iodide (PI) double-staining assay. **F** The cell migration of SW480 cells in control, OE-NC, OE-SCRAR5, shSCRAR5, and shNC groups, respectively, by Transwell assay. **G** The relative wound area of SW480 cells in control, OE-NC, OE-SCRAR5, shSCRAR5, and shNC groups, respectively, by wound healing assay

**Fig. 3:**
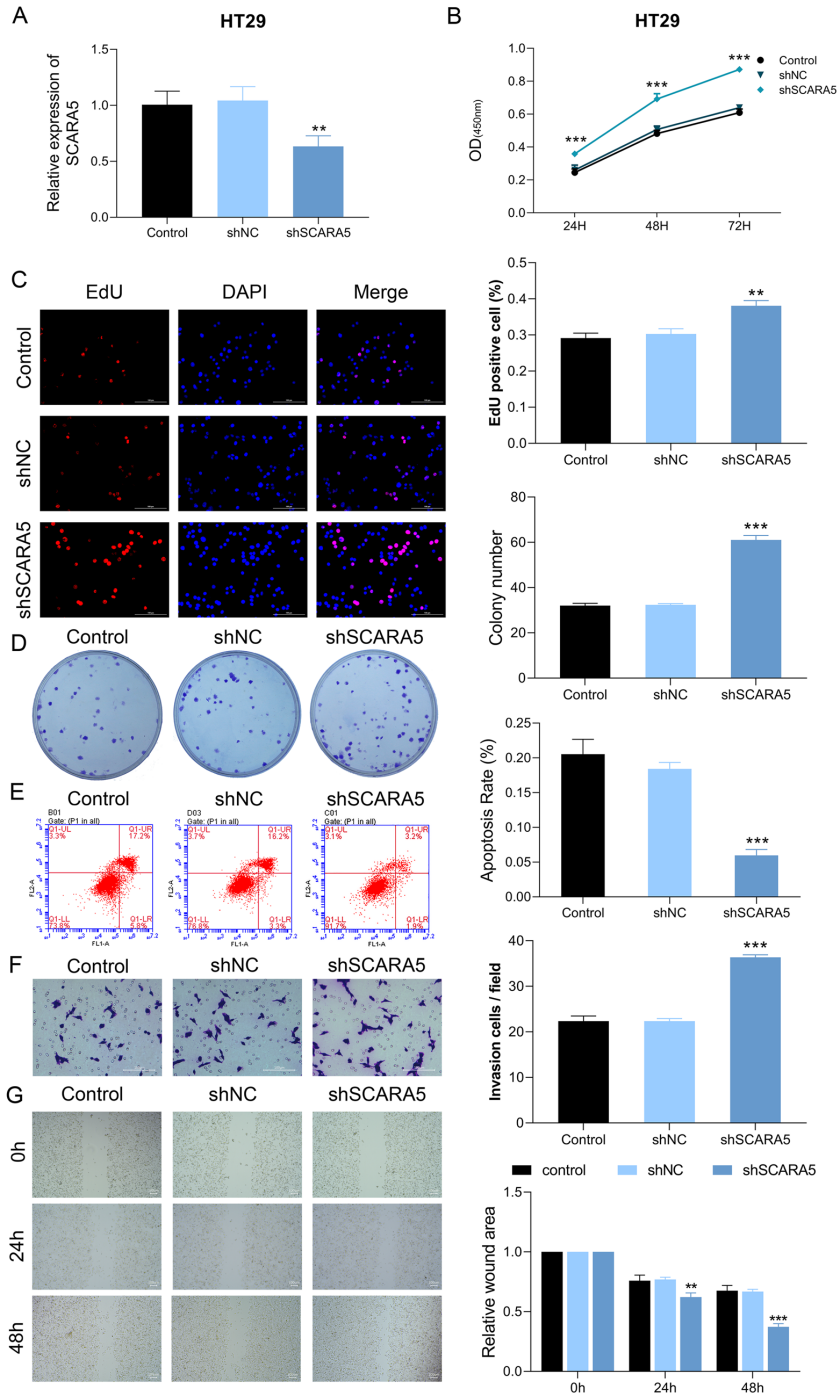
Scavenger receptor class A member 5 (SCRAR5) silence promoted cell proliferation, inhibited cell apoptosis, and increased cell migration in HT29 cells. **A** The mRNA level of SCRAR5 in HT29 cells transfected with SCRAR5 silence plasmid (shSCRAR5), and the corresponding negative control shNC, respectively. **B** Cell viability of HT29 cells in control, shSCRAR5, and shNC groups, respectively, in time-dependent manner by CCK-8 assay. **C** Cell proliferation of HT29 cells in control, shSCRAR5, and shNC groups, respectively, by5-ethynyl-2′- deoxyuridine (EdU) assay. **D** The clone number of HT29 cells in control, shSCRAR5, and shNC groups, respectively, by colony-forming assay. **E** Cell apoptosis of HT29 cells in control, shSCRAR5, and shNC groups, respectively, by annexin V-fluorescein isothiocyanate (FITC)/propidium iodide (PI) double-staining assay. **F** The cell migration of HT29 cells in control, shSCRAR5, and shNC groups, respectively, by Transwell assay. **G** The relative wound area of HT29 cells in control, shSCRAR5, and shNC groups, respectively, by wound healing assay

SW480 cells were transfected with OE-SCRAR5, OE-NC, shSCRAR5, and shNC, respectively. Compared with cells with OE-NC or shNC, the mRNA level of SCRAR5 was remarkably up-regulated in cells transfected with OE-SCRAR5, while SCRAR5 expression was significantly down-regulated in cells with shSCRAR5 (p < 0.05, Fig. 2A). Cell proliferation assays revealed that compared with control and OE-NC or siNC groups, SCRAR5 overexpression obviously inhibited cell viability in time-dependent manner (p < 0.05, Fig. 2B), decreased the EdU fluorescence (Fig. 2C), and reduced the clone number (Fig. 2D); reversely, SCRAR5 knockdown increased cell viability (Fig. 2B), the EdU fluorescence (Fig. 2C), and the clone number (Fig. 2D). In addition, SCRAR5 overexpression induced cell apoptosis, while SCRAR5 knockdown suppressed cell apoptosis when compared with control cells (Fig. 2E). Meanwhile, cell migration was also evaluated by wound healing assay and transwell assay. Compared with control and OE-NC or siNC groups, the cell migration rate was decreased and the relative wound area was increased in cells transfected with OE-SCRAR5, while which were reversed in cells with shSCRAR5 (p < 0.05, Fig. 2F, G). Furthermore, HT29 cells were transfected with shSCRAR5, and shNC, respectively. Compared with cells with shNC, SCRAR5 expression was significantly down-regulated in cells with shSCRAR5 (p < 0.05, Fig. 3A). Cell proliferation assays revealed that compared with control and siNC groups, SCRAR5 knockdown increased cell viability (Fig. 3B), the EdU fluorescence (Fig. 3C), and the clone number (Fig. 3D). Additionally, SCRAR5 knockdown suppressed cell apoptosis when compared with control cells (Fig. 3E). Compared with control and siNC groups, the cell migration rate was increased and the relative wound area was decreased in cells transfected with shSCRAR5 (p < 0.05, Fig. 3F, G).

### Effect of SCRAR5 abnormal expression on PI3K/AKT/mTOR pathway in CRC cells

**Fig. 4:**
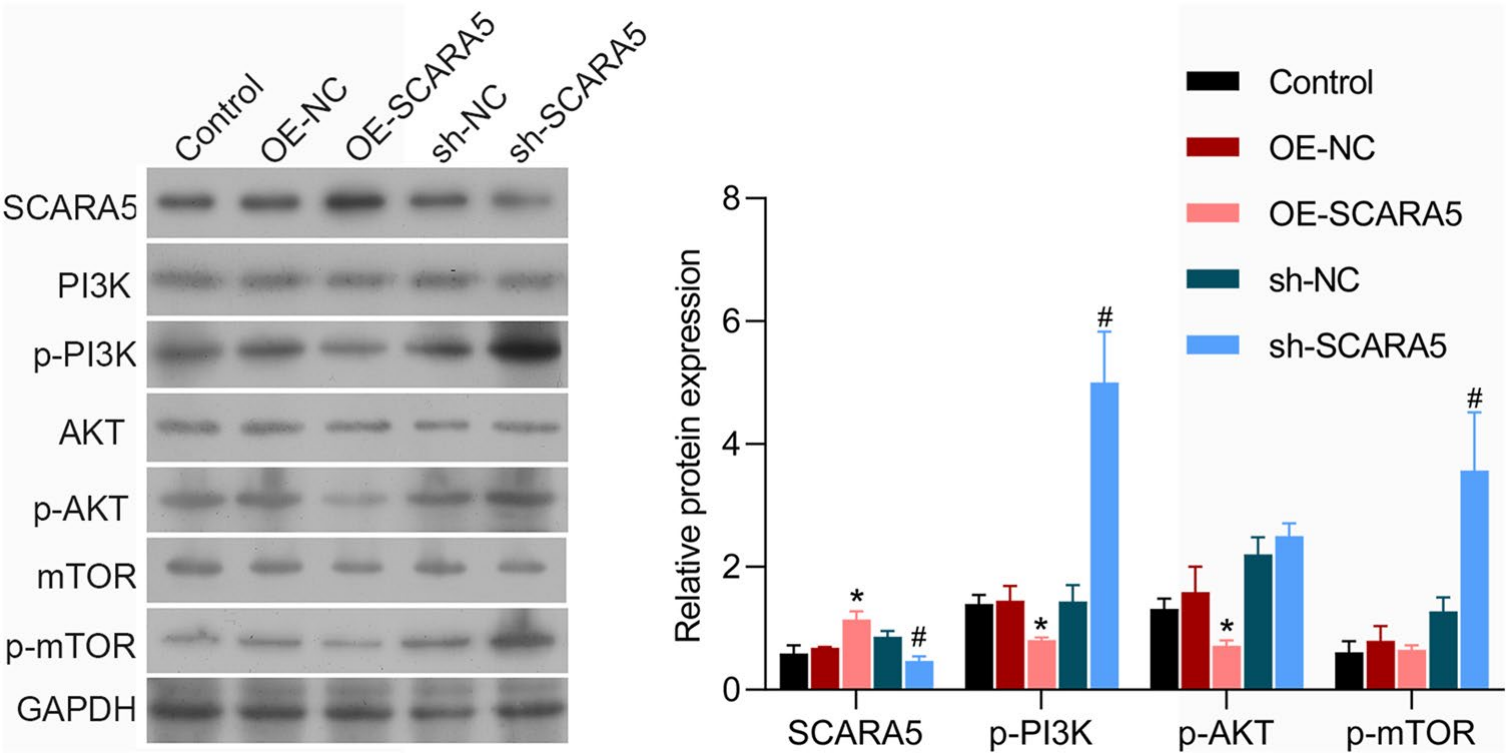
Scavenger receptor class A member 5 (SCRAR5) overexpression inhibited PI3K/AKT/mTOR pathway in SW480 cells. The protein expression of SCRAR5, phosphorylated-phosphatidylinositol-3-hydroxykinase (p-PI3K), PI3K, p-AKT, AKT, p-mTOR, and mTOR of SW480 cells in control, OE-NC, OE-SCRAR5, shSCRAR5, and shNC groups, respectively, by western blotting

Furthermore, PI3K/AKT/mTOR pathway was detected to reveal the mechanism of SCRAR5 overexpression that inhibited the growth and migration of CRC cells. Compared with cells with OE-NC or shNC, the protein level of SCRAR5 was highly expressed in cells transfected with OE-SCRAR5, while lowly expressed in cells with shSCRAR5 (Fig. 4). Interestingly, SCRAR5 overexpression decreased the expression of p-PI3K, p-AKT, and p-mTOR, while SCRAR5 knockdown promoted their expressions (Fig. 4).

### In vivo anti-tumor effect of SCRAR5 overexpression on CRC xenograft mice

**Fig. 5:**
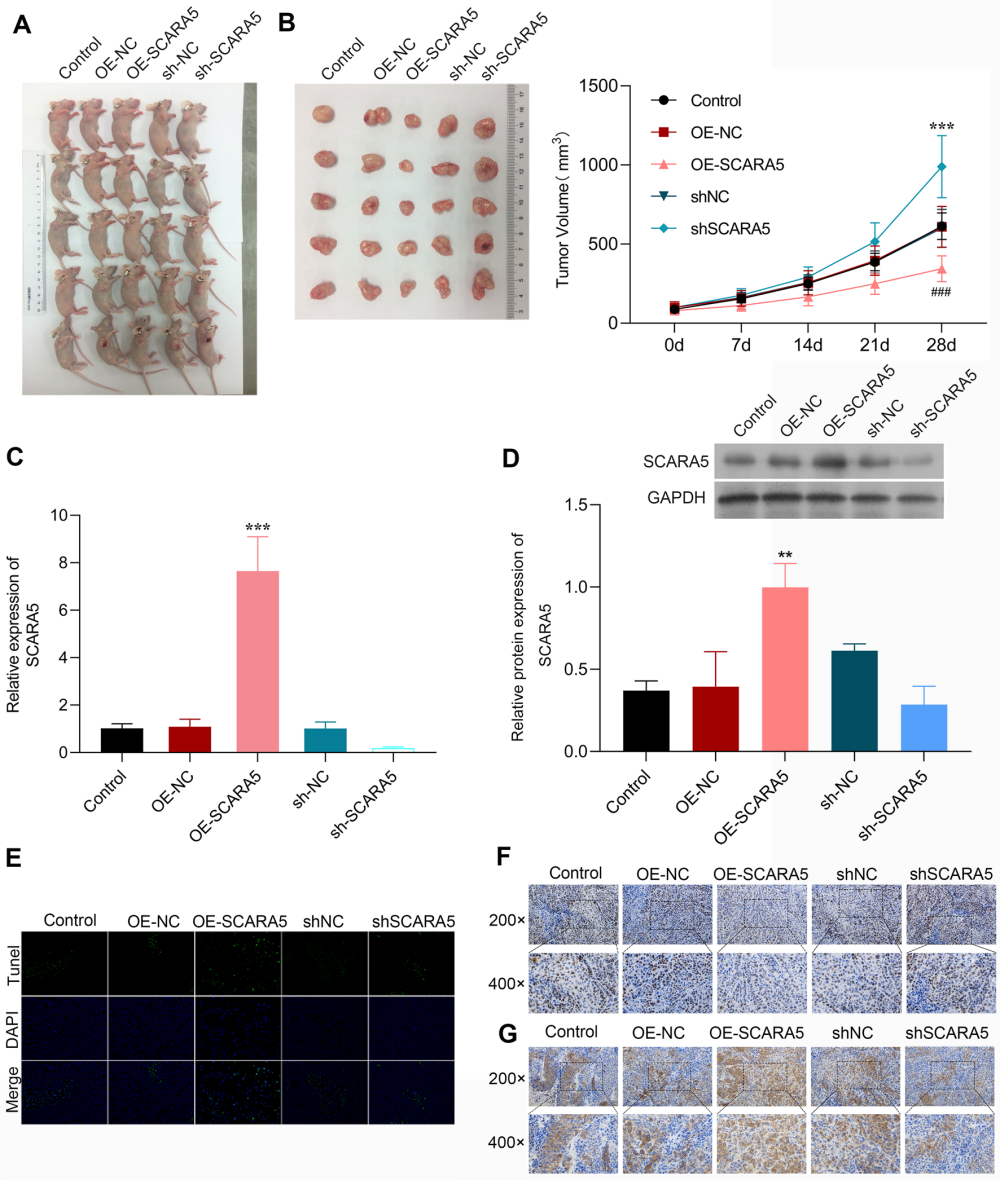
Scavenger receptor class A member 5 (SCRAR5) overexpression inhibited tumor growth in colorectal cancer (CRC) xenograft mice. **A** The tumor image of CRC xenograft mice in control, OE-NC, OE-SCRAR5, shSCRAR5, and shNC groups. **B** Tumor volumes of CRC xenograft mice in control, OE-NC, OE-SCRAR5, shSCRAR5, and shNC groups in a time-dependent manner. The mRNA (**C**) and protein (**D**) levels of SCRAR5 of CRC xenograft mice in control, OE-NC, OE-SCRAR5, shSCRAR5, and shNC groups. **E** The cell apoptosis of CRC xenograft mice in control, OE-NC, OE-SCRAR5, shSCRAR5, and shNC groups by Tunel assay. The Ki-67 (**F**) and SCRAR5 (**G**) expressions of CRC xenograft mice in control, OE-NC, OE-SCRAR5, shSCRAR5, and shNC groups by immunohistochemistry

In vivo experiments found that the tumor volumes of CRC xenograft mice were reduced in the OE-SCRAR5 group in comparison to the control or OE-NC group in a time-dependent manner (p < 0.01, Fig. 5A, B), while and the tumor volume was increased in the shSCRAR5 group (p < 0.01, Fig. 5A, B). Meanwhile, both the mRNA and protein levels of SCRAR5 were elevated after OE-SCRAR5 treatment and decreased after shSCRAR5 treatment in mice (Fig. 5C, D). Furthermore, Tunel assay revealed the increased apoptotic cells in the OE-SCRAR5 group and reduced apoptotic cells in the shSCRAR5 group compared with the control and NC groups (Fig. 5E). IHC showed that the Ki67 expression trend in various groups was consistent with the tumor volume of mice (Fig. 5F). SCRAR5 expression detected by IHC also similar to the qPCR and western blotting assays in various groups (Fig. 5G).

## Discussion

High metastasis and drug resistance to chemotherapeutic drugs have become a key factor hindering the long-term survival of patients with CRC. The abnormal expression of SCRAR5 has been reported to be involved in various cancers. However, the roles and mechanisms of SCARA5 during the occurrence and progression CRC still unclear. In the present study, we found the down-regulation of SCRAR5 in CRC cells. Importantly, SCRAR5 overexpression obviously inhibited CRC cell growth and migration, which might be related to PI3K/AKT/mTOR pathway. Consistently, in vivo experiments also revealed that SCRAR5 overexpression exerted anti-tumor effects in CRC xenograft mice.

Several studies have investigated the role of SCRAR5 in cancers, and they have shown that SCRAR5 participates in cancer progression as tumor suppressor. For example, SCRAR5 is reported to be dramatically down-regulated in breast cancer, and promote breast carcinogenesis by regulating promoter methylation (Ulker et al. 2018). Also, SCRAR5 is proposed to be lowly expressed in breast cancer, and SCRAR5 overexpression restrains the tumor proliferation and metastasis by serving as tumor suppressor (You et al. 2017). Liu et al. have shown that down-regulation of SCRAR5 aggravates hepatocellular carcinoma progression via regulating β-catenin degradation in vitro and in vivo experiments (Liu et al. 2018b). Notably, SCRAR5 is reported to involve with CRC progression (Liu et al. 2020). Similarly, this study found that SCRAR5 was down-regulated in CRC cells. Thus, we prompted that SCRAR5 might be a potential tumor suppressor in CRC.

To further uncover the role of SCRAR5 in CRC, we evaluated the role of SCRAR5 abnormal expression on cell proliferation, apoptosis, and migration in CRC cells as well as in CRC xenograft mice, and the results revealed that SCARA5 upregulation remarkedly inhibited cell growth in SW480 cells, as well as inhibited tumor growth in CRC xenograft mice, which indicated that SCARA5 participated in abnormal tumor proliferation in CRC in vitro and in vivo. Cell apoptosis is key indicator in cancer research, which can reflect the cancer progression and prognosis (Xu et al. 2019; Xue et al. 2019). Both in vitro and in vivo experiments in this study revealed SCARA5 overexpression induced cell apoptosis, thereby inhibiting tumor growth. The migration ability of tumor cells is another pivotal indicator to evaluate cell aggressiveness and tumor progression. A previous report has shown that SCARA5 knockdown contributes to epithelial-to-mesenchymal transition-induced migration in lung carcinoma A549 cells (Liu et al. 2013). The current study also found that overexpression of SCARA5 exhibits inhibiting effect on cell migration ability of SW480 cells. These findings suggested that SCARA5 play a crucial role in CRC metastasis.

To further explore the concrete mechanism on the tumor-inhibiting effects of SCARA5 in CRC, we detected the influences of SCARA5 abnormal expression in PI3K/AKT/mTOR pathway. PI3K/AKT pathway has been reported to be closely implicated in cell proliferation, apoptosis and migration (Noorolyai et al. 2019). The phosphorylation of PI3K is able to modify the protein structure of AKT and then activate AKT, which further regulates tumor cell proliferation, differentiation, apoptosis, and migration (Noorolyai et al. 2019). In addition, mTOR served as a downstream target of PI3K/AKT pathway also can be activated to involve with tumor cell progression and chemoresistance (Polivka Jr and Janku 2014). Interestingly, previous study has demonstrated that activation of PI3K/AKT/mTOR signaling pathway is beneficial to cell proliferation and migration in CRC (Narayanankutty 2019; Pandurangan 2013). Additionally, PI3K/Akt/mTOR pathway is involved in the metastatic initiation and drug resistance events of CRCs (Narayanankutty 2019). Due to the pivotal role of PI3K/Akt/mTOR pathway in CRC, more researches have focused on the natural and synthetic small molecules that can effectively target this pathway as potent inhibitors of CRCs (Bahrami et al. 2018). Fortunately, data from several clinical trials confirm the therapeutic effects of pathway inhibitors such as BYL719, KRX-0401, MK-2206, BEZ235, and everolimus in CRC patients (Bahrami et al. 2018). Consistently, our study also revealed that SCARA5 overexpression down-regulated the expression of p-PI3K, p-AKT, and p-mTOR, while SCRAR5 silence contributed to the activation of PI3K/AKT/mTOR pathway. Therefore, we speculated that PI3K/AKT/mTOR signaling pathway might participate in the effects of SCRAR5 on cell proliferation, apoptosis and migration in CRC.

In conclusion, the data of this study suggested that SCRAR5 was a potential tumor suppressor by regulating tumor growth and metastasis in CRC, which might be associated with PI3K/AKT/mTOR signaling pathway.
